# Automatic time-shift alignment method for chromatographic data analysis

**DOI:** 10.1038/s41598-017-00390-7

**Published:** 2017-03-21

**Authors:** Qing-Xia Zheng, Hai-Yan Fu, He-Dong Li, Bing Wang, Cui-Hua Peng, Sheng Wang, Jun-Lan Cai, Shao-Feng Liu, Xiao-Bing Zhang, Yong-Jie Yu

**Affiliations:** 1 Zhengzhou Tobacco Research Institute of CNTC, Zhengzhou, 450001 China; 2 School of Pharmaceutical Sciences, South-Central University for Nationalities, Wuhan, 430074 China; 3 Ningxia Engineering and Technology Research Center for Modernization of Hui Medicine, Ningxia Medical University, Yinchuan, 750004 China; 4 College of Pharmacy, Ningxia Medical University, Yinchuan, 750004 China

## Abstract

Time shift among samples remains a significant challenge in data analysis, such as quality control of natural plant extracts and metabolic profiling analysis, because this phenomenon may lead to invalid conclusions. In this work, we propose a new time shift alignment method, namely, automatic time-shift alignment (ATSA), for complicated chromatographic data analysis. This technique comprised the following alignment stages: (1) automatic baseline correction and peak detection stage for providing useful chromatographic information; (2) preliminary alignment stage through adaptive segment partition to correct alignment for the entire chromatogram; and (3) precise alignment stage based on test chromatographic peak information to accurately align time shift. In ATSA, the chromatographic peak information of both reference and test samples can be completely employed for time-shift alignment to determine segment boundaries and avoid loss of information. ATSA was used to analyze a complicated chromatographic dataset. The obtained correlation coefficients among samples and data analysis efficiency indicated that the influences of time shift can be considerably reduced by ATSA; thus accurate conclusion could be obtained.

## Introduction

Development of chromatographic fingerprint-based methods for quality control of natural plant extracts, such as essential oils, is important for medical and industrial applications. High-performance liquid chromatography and gas chromatography (GC) are efficient techniques used to collect chemical information of samples^1–3^. Manual verification is an inefficient and irreproducible task because of the complexity of chromatographic fingerprints^4–6^. Analysis of these fingerprints is rarely straightforward in practical applications. Therefore, time-shift occurrence should be considered because it may affect the accuracy of data analysis and lead to invalid conclusions.

Time-shift correction is a significant and interesting aspect of chromatographic data preprocessing^4–11^. Preprocessing methods are categorized into three types. The first and major type of preprocessing is based on warping strategy, which compresses or stretches chromatographic signals to maximally fit the target one. This category includes correlation optimized warping (COW)^12^, dynamic time warping^13^, and their variants^5, 10, 12, 14–16^. The second type of preprocessing utilizes deletion or insertion of data points to align chromatographic signals^17–20^. In practical applications, these methods are suitable for analysis of chromatograms, whose components are satisfactorily separated. The last type of preprocessing techniques analyzes two-dimensional chromatographic signals obtained from hyphenated chromatographic instruments, such as GC hyphenated with a spectrometer^21–31^. These methods are generally used to analyze profiling datasets, such as those for metabolic profiling.

In our previous study, we proposed a peak alignment method for metabolic profiling analysis (named CAMMPA)^27^. Alignment was performed for chromatographic peaks in the reference sample. Chromatographic peaks of the test sample may be eliminated if they are absent in the reference sample. In conclusion, CAMMPA is unsuitable for quality control based on an entire chromatogram. In the present work, a novel automatic time-shift alignment method (ATSA) was developed for chromatographic data analysis. The proposed method was employed for analysis of a large-scale GC chromatographic dataset to monitor the quality of essential oil.

## Theory

### ATSA

ATSA consists of three main stages, namely, (1) baseline correction and chromatographic peak detection; (2) preliminary alignment based on large segment size, in which a number of reference chromatographic peaks are eluted; and (3) precise alignment of each chromatographic-peak segment in test. Each stage will be explained thoroughly in the following in detail.

#### Baseline correction and chromatographic peak detection

Baseline drift is an essential parameter that should be considered in quality control and metabolic profiling analysis. In the present work, we introduced our recently developed method, namely, local minimum values-robust statistical analysis (LMV-RSA)^32^, to eliminate baseline drift prior to data analysis. First, LMVs in the chromatogram were extracted, and the corresponding positions were marked. An iterative optimization strategy based on RSA was utilized to remove LMVs that belong to chromatographic peaks. Finally, baseline drift was estimated using linear interpolation.

Time shift refer to chromatographic peaks. An automatic chromatographic peak detection strategy^33^ was used in the developed method. Peak detection was performed based on multi-scale Gaussian smoothing strategy, which was established based on the fact that chromatographic peaks are local maximal values and will be maintained under various Gaussian smoothing scales. Thus, chromatographic peaks could be readily recognized based on the ridge lines. Moreover, useful chromatographic information, involving retention time, peak elution range, peak height, peak area, can be obtained.

#### Preliminary alignment

Time shift situations may differ among various elution ranges. The entire chromatogram should be divided into a number of segments^18^. Preliminary alignment stage focuses on correcting the time shift of each segment.

First, the reference chromatogram should be provided. In this work, the chromatogram with highest correlation coefficient was selected as reference chromatogram. Time-shift value is another parameter that should be estimated, and a shift value of 0.5 min could be employed for most situations.

**a**) **Initialization of segment size**. Our experience indicated that time-shift value could be similar within a short elution range, such as 2 or 3 min. In the present work, a 3 min segment size was used. If the retention time distance between the current and first peaks in the segment is less than 3 min, then this peak will be arranged into the same segment; otherwise, a new segment will be added and the current peak will treated as the first one in this added segment. A segment with less than three chromatographic peaks will be syncretized with a neighbor segment with fewer peaks. Finally, fourteen segments were obtained for the reference chromatogram.

If all chromatographic peaks are adequately arranged, then the start and end boundaries of a segment are temporarily treated as the beginning position of the first peak and the end position of the last peak in the segment, respectively. The boundaries will be modified as the average value of two successive segments to ensure that the end boundary of the segment is the same as the start boundary of the following one.

Boundaries of a test segment were determined by combining those of the corresponding segment in the reference with the pre-estimated time-shift value (in this work, the time-shift value was set as 0.5 min). The size of the test segment is larger than that of the reference segment by 1 min. For example, the start and end boundaries of the second segment of the reference were 6.36 and 9.58 min, respectively, whereas those for the corresponding test segment are 5.86 and 10.08 min, respectively. In the preliminary segment procedure, the boundaries of the test segment are the search space of the corresponding reference segment.

**Figure 1:**
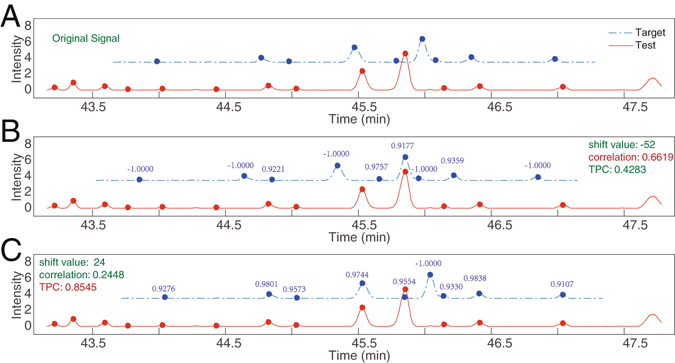
Illustration of difference between correlation coefficient and total peak correlation coefficient (TPC). (**A**) Original reference and test chromatograms with chromatographic peaks by circle. (**B**) Aligned chromatogram with maximal Pearson product-moment correlation coefficient. (**C**) Obtained aligned chromatogram with maximal TPC.

**b**) **Alignment criterion**. In the alignment procedure, an alignment criterion should be selected to maximally match the test chromatogram with the reference. Most of the current alignment methods employ the Pearson product-moment correlation coefficient criterion, which can be calculated as follows:

1 $$c=\frac{{({\boldsymbol{r}}-\bar{r})}^{T}({\boldsymbol{x}}-\bar{x})}{\sqrt{{({\boldsymbol{r}}-\bar{r})}^{T}({\boldsymbol{r}}-\bar{r}){({\boldsymbol{x}}-\bar{x})}^{T}({\boldsymbol{x}}-\bar{x})}}$$

where *c* is the correlation coefficient; vector ***r*** is the reference chromatogram; vector ***x*** is the test chromatogram; and $\bar{r}$ and $\bar{x}$ are the mean values of vectors ***r*** and ***x***, respectively. The correlation coefficient can be directly and efficiently calculated. However, correlation coefficient pays more attention on large values. If an extremely large chromatographic peak is present only in either the reference or test segment, the correlation coefficient criterion may lead to inadequate results for small peaks. Figure 1 provides an example based on the correlation coefficient criterion. Figure 1A shows the original chromatogram, and Fig. 1B depicts the aligned chromatogram with the maximal correlation coefficient (0.6619); the largest peak in the reference chromatogram was aligned to the largest one in the test chromatogram. However, the remaining chromatographic peaks in the reference chromatogram were misaligned. Although the aligned chromatogram in Fig. 1C was more reasonable than that in Fig. 1B, the obtained correlation coefficient (0.2448) is lower than that in Fig. 1B.

Peak based correlation coefficient provides another criterion. First, the peak positions in the reference and test were marked. If a test peak is absent in the elution range of the reference peak, the reference peak correlation coefficient was manually set as −1.0000. Figure 1B shows that five of the nine reference peaks did not match any peaks in the test sample. For each of the matched peak, time shift was corrected by retention time, and the elution range in the test was temporally set the same as that of the reference peak. Correlation coefficient was then calculated according to Equation (1). Figure 1B shows the correlation coefficient for each matched peak. Finally, total peak correlation coefficient (TPC) was calculated as:

2 $${\rm{TPC}}=(\sum _{i=1}^{I}{w}_{i}\ast {c}_{i}/\sum _{i=1}^{I}{w}_{i})\ast (I/N)$$

3 $${w}_{i}=PeakAre{a}_{i}/PeakLengt{h}_{i}$$

where *w*_*i*_ is the weight for the *i*th-matched peak and calculated according to Equation (3); *c*_*i*_ is the *i*th-peak correlation coefficient; *I* is the number of matched peaks and equal to 4 in Fig. 1B; *N* is the number of peaks in the reference chromatogram and equal to 9 in Fig. 1B. The *PeakLength*_*i*_ is the number of points contained in a peak, which is calculated as *end point* - *start point* +1.

Figure 1B and C show TPCs under different shift values. The aligned chromatogram in Fig. 1C is more reasonable because chromatographic peaks have been adequately aligned. Time-shift alignment primarily aims to provide reasonable results for chromatographic peaks, as such, we utilized TPC as the alignment criterion in our method.

**Figure 2:**
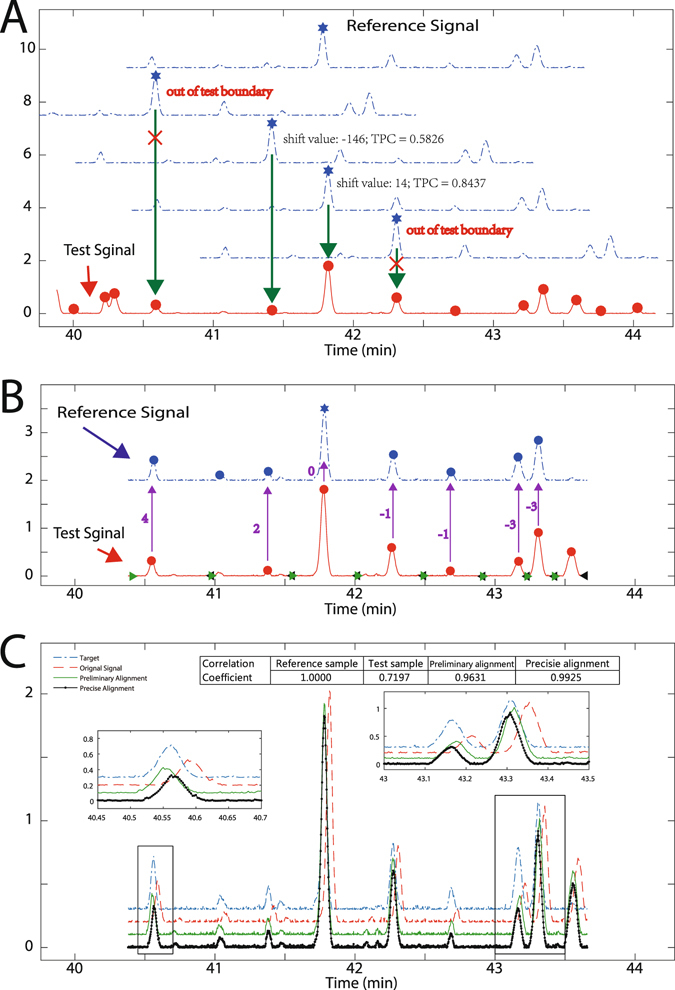
Illustration time shift alignment of the ATSA. (**A**) Candidate peaks in the test segment. (**B**) Precise alignment based on the segment of test chromatographic peaks. (**C**) Aligned chromatograms. Values in (**B**) indicated time-shift values for precise alignment.

**c**) **Peak-to-peak-based alignment**. In the present work, a peak-to-peak alignment strategy was developed to perform time-shift correction for each segment by using the constraint that the boundary of the reference chromatogram should not exceed that of the test chromatogram. Figure 2A shows an example of the peak-to-peak-based alignment strategy, which was performed based on the largest peak (marked by star) in the reference. Only two alignments were needed to be performed, i.e. −146 shift value and 19 elution channels (elution channel is the scanning point in the chromatogram). The remaining alignments should never be implemented because they exceeded the boundary of the test chromatogram. In each alignment, TPC was calculated, and the time shift of 19 elution channels with a larger TPC value seemed to be acceptable (Fig. 2A).

**d**) **Modification of incorrect alignment**. In the preliminary alignment, some segments may not be adequately aligned, i.e. the shift values of the segments differ from those of the other segments. These incorrectly aligned segments can be treated as outliers. A robust statistical method was then used to detect inadequately aligned segments:

4 $$\sigma =1.483\ast \mathop{{\rm{median}}}\limits_{i=1,\ldots ,J}|{s}_{i}-\mathop{{\rm{median}}}\limits_{j=1,\ldots ,J}({s}_{j})|$$

5 $${d}_{k}=|{s}_{k}-\mathop{{\rm{median}}}\limits_{j=1,\ldots ,J}({s}_{i})|/\sigma $$

where *s*_*k*_ is the time-shift value of the *k*th segment; *median* is the median value of all the time-shift values for all *J* segments; |·| is the absolute value; *σ* is the median absolute deviation; the parameter 1.483 is the adjustment factor that makes the *σ* belong to the normal distribution^34^. Time-shift value of the *k*th segment is treated as outlier when |*d*_*k*_| is larger than 2.5 (99% confident level).

If a segment is judged as an outlier, then time-shift alignment should be re-performed. In this case, chromatogram was aligned along the elution channel within ±2.5*σ* time-shift range. The TPC alignment criterion was abandoned, and the correlation coefficient was used to improve the alignment efficiency. Local maximal correlation coefficient values were recorded. The coefficient with the smallest distance to the expected time-shift value, i.e., $\mathop{{\rm{median}}}\limits_{j=1,\ldots ,J}({s}_{j})$, will be selected as the optimal one. Finally, time-shift value for each segment in the test chromatogram could be obtained using the boundaries of the segments.

**e**) **Warping strategy**. When all segments are adequately aligned, the boundaries of each segment could unavoidably overlap or disconnect with its neighbors. In this work, a warping strategy was employed. First, the boundary of each segment was modified. In segment disconnection cases, the boundary could be simply treated as the average value in two successive segments. For instance, the end positon of the 12th segment is 14833, and the start position of the 13th segment is 14839 after time shift correction. After modification, both values were modified to be 14836.

In the cases of overlapped boundary situations, modification may be complicated, especially when the start and/or end position of a chromatographic peak is within the overlapped zone. If the peak end position is nearer the middle of the overlapped zone, then the start boundary of the later segment will be modified and the boundary will be set as the average value between the end boundary of the former segment and the start boundary of the subsequent segment. By contrast, if the peak start position is nearer the middle of the zone, then the end boundary of the former segment will be modified. Otherwise, the boundary will be modified as the average value.

The length of the test segment should be equal to that of the reference segment. Results indicated that only 3 (seventh, ninth, and eleventh segments) of the 14 segments maintained their original length after alignment. Eight segments became longer after time-shift alignment. To ensure that the test chromatogram keeps the same length as the reference chromatogram after preliminary alignment, we adopted a warping strategy based on linear interpolation, which compresses long segments and stretches short segments. In the linear interpolation stage, the start and end position was fixed to generate a new vector with linearly equally spaced points between the start and end position. The original position and signal were then used to build the linear model in MATLAB. The new vector was used to model the preliminary-aligned chromatogram.

In the preliminary alignment, peak information in the test chromatogram, involving peak start and end position as well as retention time, will be modified accordingly.

#### Precise alignment

The preliminary alignment could maximally correct time shift for each segment. However, the time-shift problem of small peaks persists because it slightly differs in the elution range of each peak. To eliminate these artifacts, we performed precise alignment.

**a**) **Chromatographic peak based partition**. The aligned test chromatogram was divided into a number of sub-segments based on chromatographic peaks involved. The boundary of each sub-segment is the average value between the end position of the chromatographic peak and the start position of the following peak. Figure 2B shows an example of eight chromatographic peaks, where the boundaries of successive segments are connected among one other.

**b**) **Peak-based precise alignment**. Each sub-segment was directly aligned to the nearest reference peak by retention time. The reference peak should be eluted within the elution range of the test chromatographic peak in this sub-segment. For instance, Fig. 2B shows the shift value for each sub-segment. If the corresponding reference peak is absent, then the shift value was estimated as the average of the neighboring sub-segments. For example, the last segment in Fig. 2B did not find a reference chromatographic peak, but its time-shift value was finally estimated as −2 elution channel, which is the mean value of −3 (shift value of the left neighbor sub-segment, Fig. 2B) and −1 (shift value of the right neighbor sub-segment, data not shown).

**c**) **Warping strategy**. As indicated in Fig. 2B, the precise shift value differs among segments. The boundaries of sub-segments will overlap or disconnect as well, which are similar to those of the preliminary alignment. Therefore, warping was utilized. First, the boundaries of each sub-segment were modified as the average value between two successive boundaries. Each sub-segment was separated into left and right parts according to the retention time position. Linear interpolation was used to compress or stretch each sub-segment part independently. In this precise alignment, the retention time of each peak will be precisely located after time-shift correction and the problem across samples can maximally corrected.

Figure 2C shows a part of the aligned chromatogram after precise alignment. The original test chromatogram and preliminary aligned result were provided for comparison. The inserted plots in Fig. 2C indicated that the time-shift problem persisted after preliminary alignment. These slightly shifted values could be accurately modified after precise alignment. The correlation coefficient value between the reference and original test chromatogram is 0.7197, which implied that the test chromatogram might belong to an unqualified sample. However, the correlation coefficient value (0.9631) was significantly improved after the preliminary alignment. Finally, the highest correlation coefficient after precise alignment, i.e., 0.9925, confirmed that this test chromatogram belongs to a qualified sample.

### COW

The famous COW was firstly developed by Nielsen *et al.* ^12^ and then modified by Tomasi *et al.* ^13, 20, 35^. This strategy has been successfully used for chromatographic analysis in many scientific fields. Prior to data analysis, the entire chromatogram should be divided into a number of segments, and the slack value should be pre-estimated for COW^35^. The ideologies and advantages of COW were illustrated by Tomasi *et al.* ^13, 35^.

## Results and Discussion

### Time-shift alignment results

**Figure 3:**
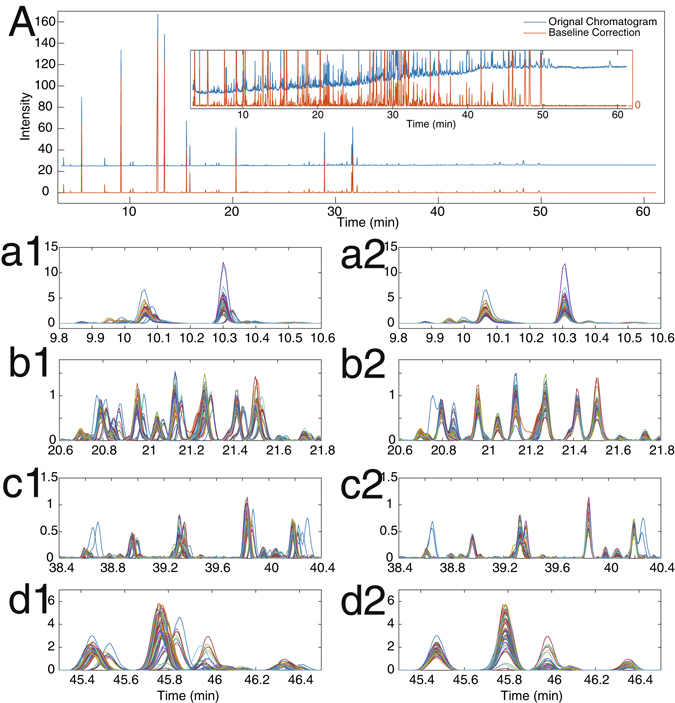
(**A**) Reference chromatograms with and without baseline correction. Left column: Original chromatograms without time-shift alignment. Right column: Chromatograms with time-shift alignment.

Figure 3A provides the original reference chromatogram and the baseline-corrected chromatogram, in which more than 200 chromatographic peaks were detected. In complicated sample analysis, such as quality control of essential oil and non-targeted metabolic profiling, a complex chromatogram is remarkably common. The experiments may not be accomplished within a few days; hence, baseline-drift and time-shift problems are inevitable.

Figure 3 The left column of Fig. 3 shows the time shift across samples. Figure 3a1 shows an elution range with two larger peaks and a number of relative smaller peaks. Figure 3b1 depicts an elution range, where more than 10 chromatographic peaks were eluted within 1.2 min; this phenomenon may occur in an extremely complicated sample analysis. Figure 3c1 shows an example where most chromatographic peaks have been separated, except for the first peak and last peak in the two test chromatograms. The overlapped peaks may promote time-shift alignment methods to provide misaligned chromatograms. Figure 3d1 presents chromatographic peaks with satisfactory separation and ideal peak shape. These peaks are frequently encountered in quantitative analysis. However, the presence of time shifts may lead to random selection of an incorrect peak for quantification; hence, invalid conclusions may be obtained.

The right column of Fig. 3 shows the corresponding time-shift correction results by the developed method, ATSA. In contrast to the profiles in left column, the time-shift problem across samples has been satisfactorily corrected. Specifically, Fig. 3a2 shows the separation of the small peaks from two larger peaks. Chromatographic peaks in Fig. 3b2 can be easily detected and adequately aligned. Figure 3c2 indicates that the developed method can successfully deal with overlapping chromatographic peaks. With the aid of time-shift alignments, each chromatographic peak in Fig. 3d2 can be accurately located. Figure 3 implied that ATSA is applicable for complex sample analysis.

### Influences of initial segment size and time shift value

**Figure 4:**
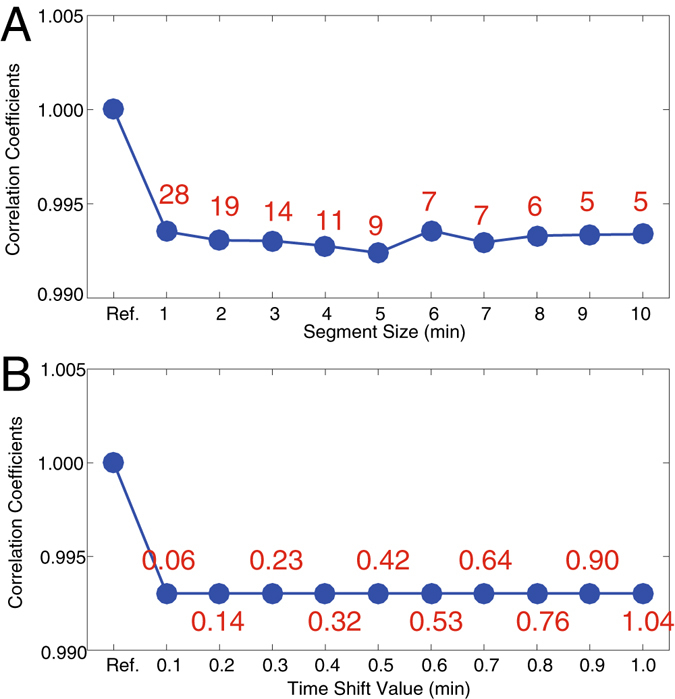
(**A**) Correlation coefficients with various initial segment sizes. (**B**) Correlation coefficients with various initial time-shift values. Values in (**A**) are the number of the final segments for preliminary alignment with different segment size. Values in (**B**) are time consumed for alignment (unit/second).

Two parameters, namely, initial segment size and time-shift value, should be pre-estimated for the developed method. In this section, the influences of initial segment size and time-shift value were investigated. Figure 4A provides the correlation coefficients under various segment sizes (initial time shift-value is 0.5 min). The coefficients varied from 0.9920 to 0.9935, indicating that the initial segment size is consistent with segment size. However, the number of final segments changed significantly. For instance, in the case when 1 min of elution was initialized, the preliminary alignment obtained 28 segments. By contrast, if the initial segment size was set as 10 min, only five segments could be obtained. The use of less number of segments (with larger segment size) in the alignment could lead to efficient analysis. However, a large segment size for preliminary alignment is not recommended, because the time-shift values of the involved peaks may considerably change, thereby increasing the level of risks for providing incorrect aligned results. In the proposed method, the default initial segment size is 3 min.

Figure 4B shows the influence of the initial time-shift value (initial segment size was 3 min). The correlation coefficient is constant and equal to 0.9921. The results indicated that ATSA is stable with respect to time-shift values. Figure 4B also shows that using small time-shift values would mean less time for data analysis. For example, only 0.06 second will be consumed for alignment if the time-shift value is set as 0.1 min. If the time-shift value is 1.0 min, then 1.04 seconds will be need, which is almost 17 times higher. In practical applications, the time shift value of 0.5 min is suitable for most situations. Therefore, 0.5 min was set as default value in time shift-alignment. Figure 4A suggests that ATSA is stable with respect to both initial segment size and time shift value, which will benefit practical applications and render ATSA as a partial method.

### Influences on quantitative results

**Figure 5:**
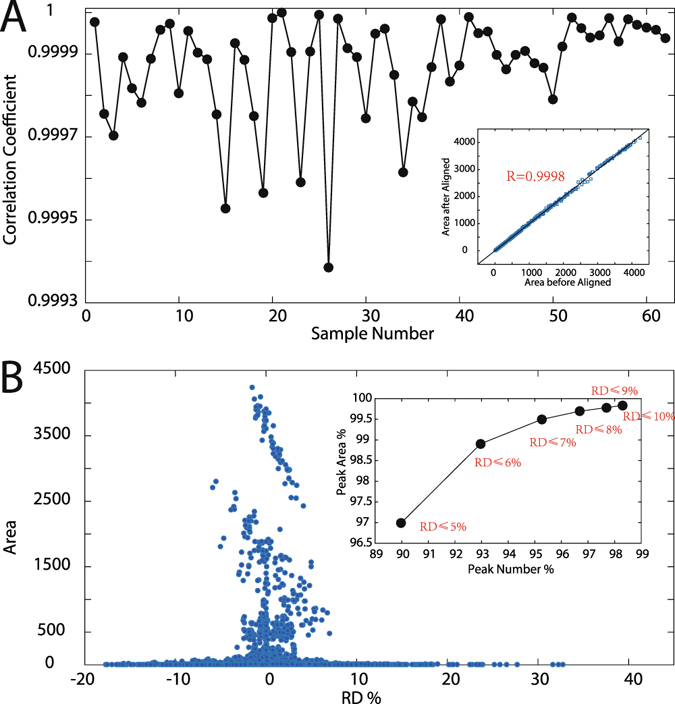
(**A**) Correlation coefficients calculated based on peak area of original and time-shift alignment chromatograms. (**B**) Relative area deviation (RD%) vs area. Inserted plot in (**A**) shows the relationship between original chromatographic areas and those after time-shift alignment. Inserted plot in (**B**) shows statistical parameter under various RD%. For instance, the marker RD% of 5%, the *x* axis indicated that 95% peaks with RD% is no more than 5%, and the value in *y* axis indicate that their area is 97% of the total area. RD% = (Area_aligned_ − Area_original_)/Area_original_ × 100%.

In complex samples analysis such as metabolic profiling analysis, data analysis are analyzed based on quantitative results. In quality control applications, chromatographic peaks in a chromatogram are definitely more important than those of instrumental noise. The influences on quantitative information, such as peak area, should not be neglected because a warping strategy was employed. Figure 5A provides the correlation coefficients between the original peak area and those after time-shift alignment. The influence could vary among different samples. However, all coefficient values are larger than 0.9993, which implies that time-shift correction will not influence quantitative information based-data analysis such as quality control of the entire chromatogram. The inserted plot in Fig. 5A provides original peak areas of all chromatographic peaks in this dataset (more than 12000 peaks) and those after the alignment. The correlation coefficient is 0.9998, which confirms that ATSA can maximally maintain quantitative information in time-shift alignment.

Figure 5B shows the changes in the peak area in a statistical manner, where relative deviation (RD) was employed. RD was calculated as: (Area_aligned_ − Area_original_)/Area_original_. Each circle in Fig. 5B marks a peak area and the corresponding RD%. The RD% of most peaks is no more than 7%. Inserted plot in Fig. 5B indicates the percent of 95% chromatographic peaks with RD% no more than 7%; the percentage of these peak areas reaches 99.5%. In the case of RD ≤10%, more than 98% chromatographic peaks were extracted and the corresponding percentage is 99.8%. Figure 5B implies that most chromatographic peak areas will change with acceptable level (7%). Large area changes in Fig. 5B usually correspond to some extremely small peaks, whose signal to noise ratio is close to the instrumental noise level that it cannot be accurately quantified in practical applications.

Figure 5 indicates that ATSA will not influence the quantitative results but correct baseline-drift and time-shift problems simultaneously. These findings will be beneficial for researchers to make valid conclusions.

### Advantaged of time shift alignment strategy

**Figure 6:**
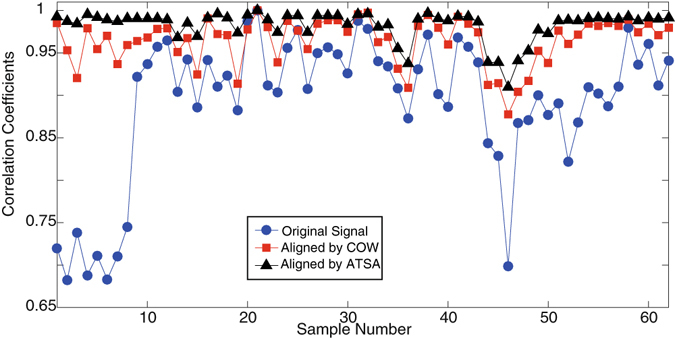
(**A**) Correlation coefficients of aligned chromatograms by COW and ATSA.

The artifacts of time shift can lead to serious problems in quality control based on the entire chromatogram. Figure 6 shows the correlation coefficients between the test and the reference samples from the original chromatograms. Several samples, including the first eight samples and the 46th sample, obtain relative low coefficients. Additionally, the coefficients of most samples are less than 0.95, which suggests that chemical composition changed during store; hence, the essential oils may be judged as unqualified samples.

The correlation coefficients significantly improved after time-shift corrected by ATSA, and only five samples with coefficient less than 0.95 were detected, which delayed in the post procedure. The quality of the studied essential oil sample is considerably stable during store procedure. However, this conclusion contradicts the results obtained based on original chromatogram; hence, the influence of time shift must be carefully addressed in practical applications.

COW, is a well-known method for time shift alignment, provides most acceptable aligned results as long as optimized parameters slack size and segment length were employed. Figure 6 shows the correlation coefficients obtained from COW. These values are a little smaller than those from ATSA. The inserted plot in Fig. 6 provides the difference between ATSA and COW. Among 62 samples, 58 samples present larger coefficients from ATSA. It seems that ATSA performs slightly better than COW.

**Figure 7:**
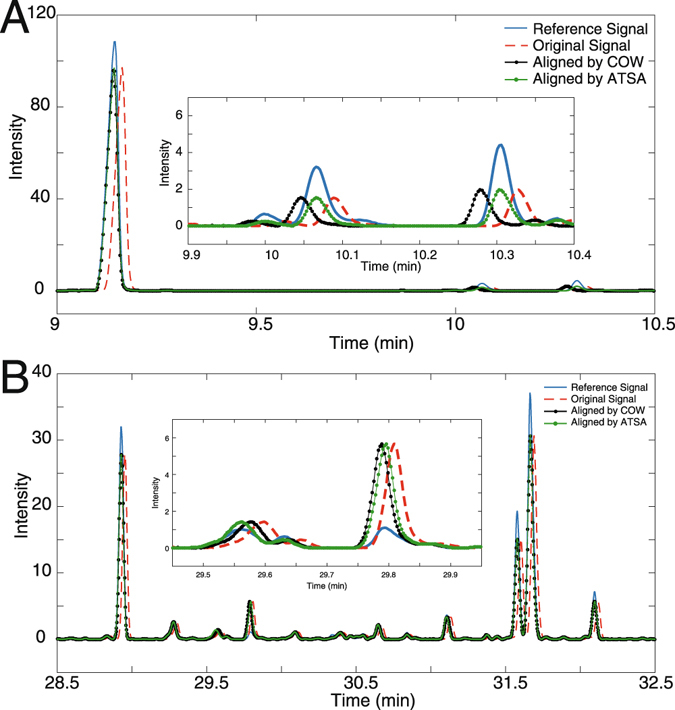
Detailed difference between COW and ATSA in the aligned chromatograms. (**A**) Elution range from 9 to 10.5 min. (**B**) Range from 28.5 to 32.5 min.

Figure 7 shows the difference between ATSA and COW by using the first test chromatogram as an example. Two elution ranges are depicted in Fig. 7. Time-shift problem in Fig. 7A and B were greatly reduced by both COW and ATSA. Evidently, the segment-based time-shift alignment can provide optimized results for the entire signal. However, time shift might be slightly different among segments, as visualized in the inserted plots in Fig. 7. Additionally, time-shift alignment usually emphasizes larger peaks in the segment and may sacrifice small peaks to obtain the highest correlation coefficients. Time shifts for larger peaks in Fig. 7 have been satisfactorily aligned, whereas those of small peaks seem persists. Figure 7 indicates that a further step for performing time shift is valuable. Fortunately, ATSA provides an option in complex chromatographic data analysis.

It should be noted that the current method, ATSA, can be treated as a direction extension of COW, because the alignment strategy of ATSA has no theoretically difference with that of COW, except that a criterion based on chromatographic peaks and a peak-to-peak alignment strategy have been employed. In fact, the advantage of ATSA compared with COW is that automatic processing procedures such as background drift correction and peak detection have been adopted. The performance of COW could be also significantly improved when these procedures have been implemented.

Figure 7 The efficiency of data analysis is a parameter that should not be neglected in a large-scale data. Less than 12 seconds are generally consumed for the entire data analysis. Considering that the studied dataset contains 62 × 23000 (sample × elution channel) data points, ATSA can be treated as an efficient method for performing time-shift alignment in complex sample analysis.

Time shift is a serious problem in chromatographic data analysis. Time-shift alignment mainly aims to provide comparable information across samples. Thus, this procedure is valuable to provide the aligned results for chromatographic peaks. In this regard, chromatographic peak information must be incorporated in the alignment to improve the aligned results.

## Conclusion

A novel time shift alignment method, namely, ATSA, was developed in this work. This method can be treated as a variant of the well-known COW by using chemical peak information for time-shift alignment because the manually depended parameters, such as chromatographic peak information, segment size, are automatically optimized in ATSA. Data processing, background-drift correction and time-shift alignment can be simultaneously performed, which is almost an automatic method. The results from essential oil samples indicated that the time shift across samples can be accurately aligned using ATSA, whose performance is comparable with that of the well-known COW. In conclusion, ATSA is an efficient approach for chromatographic data analysis.

## Experiment

### Sample Collection and preparation

This experimental was designed to verify the quality change of essential oils in different zones of China. Sixty-two samples from a commercial essential oil were collected and stored for 10 months in three cities, namely, Guangzhou, Zhengzhou, and Changchun in China. For each city, 20 samples were stored at a cool, dry place away from light, and two samples were analyzed monthly. Reference 2 samples were stored in the laboratory. All samples were posted to our laboratory for analysis. Briefly, 1 g of the sample was subjected to methyl esterification by adding 45 mL of extraction reagent (a mixture of methanol and sulfuric acid, 95/5, v/v). The solution was then placed into a water bath at 60 °C for 2 h. Subsequently, 10 mL of the methyl esterification solution was placed in a separating funnel and added with 20 mL of H_2_O and 10 mL of CH_2_Cl_2_ (JT Baker, USA). Finally, 10 mL of CH_2_Cl_2_ was transferred into a conical flask and added with 3.5 g of anhydrous sodium sulfate.

### Instrumental Condition

The solution (1 μL) was analyzed on an Agilent GC coupled with a flame ionization detector (FID). An Agilent DB-5MS (50 m × 0.25 mm, 0.25 µm) chromatographic column was used with a 1:20 split ratio of the injector at 280 °C. Helium carrier gas was used at a constant flow rate of 2.0 mL min^−1^. The temperature of the FID was set at 280 °C, and the hydrogen and air flow rates were 40 and 400 mL min^−1^, respectively. The column temperature was maintained at 45 °C for 2 min and then increased to 280 °C at a rate of 6 °C min^−1^ for 20 min. More than 23,100 × 62 data points were collected.

### Data analysis

All calculations were conducted using MATLAB 2013b (MathWorks, USA) in a computer with Windows 7 (64-bit), Intel® Core™ i5 CPU (2.8 GHz), and 8G RAM. The programs of our MATLAB program can be freely obtained from the authors on request. The MATLAB code of COW was downloaded from http://www.models.life.ku.dk/dtw_cow.
